# A Comparative Study of Parent and Child Perspectives on Using the Intranasal Mucosal Atomization Device for Behavior Management in Pediatric Dental Sedation

**DOI:** 10.7759/cureus.58832

**Published:** 2024-04-23

**Authors:** Nandini Devi M, Maria Anthonet Sruthi

**Affiliations:** 1 Department of Pedodontics and Preventive Dentistry, Saveetha Dental College and Hospitals, Saveetha Institute of Medical and Technical Sciences (SIMATS) Saveetha University, Chennai, IND

**Keywords:** mucosal atomization device, behavior, conscious sedation, pediatric sedation, intranasal sedation

## Abstract

Background

Dental anxiety in children often necessitates sedation for effective treatment. Different methods of sedation have proved to be beneficial. However, intranasal sedation provides a needleless, rapid drug delivery method that bypasses hepatic metabolism. Intranasal sedative drugs can be delivered using a mucosal atomization device (MAD). This study aimed to find the opinions, beliefs, and existing knowledge of parents and children regarding intranasal sedation and the method of drug delivery through MAD.

Materials and methods

The study comprised 50 parents, aged 20 to 50, who came in with a single child, aged five to nine years. In separate rooms, parents and kids were each shown a video about the use of the intranasal sedation technique with MAD as a pharmaceutical help during routine dental procedures. After the video presentation, each participant was required to complete a pretested self-made questionnaire with 21 questions and multiple-choice options. The chi-square test was used in the statistical analysis of the completed questionnaires (P < 0.05). Paired t-test was used for behavior assessment of the children before and after intranasal sedation.

Results

A significant correlation between parental socioeconomic status and acceptance of intranasal sedation was observed. Parents with higher education levels exhibited a greater level of acceptance (P = 0.000). Parents whose children had prior dental experiences were more likely to view intranasal sedation as a safe option (P = 0.038). Additionally, a significant proportion of previously sedated children expressed willingness to undergo treatment using intranasal sedation. Evaluation of children's behavior before and after treatment revealed a notable improvement, indicating the effectiveness of intranasal sedation (P = 0.000).

Conclusion

The study thoroughly investigated how parents and children view intranasal sedation via MAD. It revealed a positive perception of safety and trust among parents regarding this method for pediatric dental procedures.

## Introduction

Due to their fear of dental procedures, a large number of children who see the dentist are restless and anxious. In order to provide children with safe, effective, and enjoyable dental care; distinct negative behaviors, such as dread of the unknown, fear of parental separation, anxiety, shyness, and resistance, should be treated differently during dental visits. Consequently, the core of pediatric dentistry treatment is behavior control [1,2]. The development of a positive relationship between the dentist and the child is crucial for the treatment to be successful, whether in terms of the child cooperating with the procedure or adhering to the dentist's prevention recommendations. The American Academy of Paediatric Dentistry (AAPD) suggests a variety of non-pharmacological and pharmaceutical treatments [3]. They are meant to make it possible for the dentist to provide a young child who is being recalcitrant with quality oral healthcare. The methods help children feel less fearful and anxious, lead them to cooperate, and cultivate a positive attitude toward dental care. When non-pharmacological approaches fail to control a child's behavior during dental treatments, pharmacological strategies, such as sedation, are used [3,4]. Due to its lower cost and reduced risk, conscious sedation is frequently preferred over general anesthesia; however, the success of this alternative depends on the specific medicine and delivery technique used [4].

There are many ways to administer medications, each having benefits and drawbacks, including oral, nasal, intravenous, and inhalation approaches [5]. Due to its rapid onset of effect and relatively straightforward administration approach, the intranasal route is becoming more and more popular [6]. Nitrous oxide-oxygen sedation, also known as “laughing gas,” is supported by the Council of European Dentists as the recommended standard sedation technique [7,8]. It offers rapid onset and recovery, dosage flexibility, and less harm to the body's systems [7]. Intranasal sedative drugs, when delivered through a mucosal atomization device (MAD, BVM Meditech Pvt Ltd, New Delhi), offer rapid absorption into the systemic circulation via the nasal mucosa. This method stands out for its swift action and avoidance of hepatic metabolism, making it a promising alternative for effective sedation in pediatric dental procedures. One significant benefit of intranasal sedation is that its onset period is three times shorter than that of oral sedation [9]. This is brought on by the drug's quick absorption, which avoids first-pass hepatic portal metabolism. Despite the fact that pediatric dentists prefer the oral method of administration, conflict, and frustration frequently result when children refuse to take the sedative. Orally administered medication may occasionally cause children to vomit or regurgitate despite efforts to mask the frequently bitter taste [10]. Administration of a sedative agent should always be done by an anesthetist or a qualified sedation provider. The decision on the ideal route of administration, and selection of the appropriate sedative drug of choice is quite controversial. While a specific route of drug administration may have its drawbacks, the same drug administered through an alternative route presents distinct advantages. Oral sedative drug formulations like midazolam have lower tolerability in patients. Masking the bitterness of the sedative agent by mixing them with flavored syrups or water showcased increased cariogenic potential with no significant difference in acceptability. The nasal route skips the taste factor. Intranasal midazolam, on the other hand, was described as unpleasant, painful, and poorly tolerated by other authors. Low patient tolerance was caused by the injectable solution's burning sensation on the nasal mucosa [11]. However, according to several other authors, administering medication via the nasal route was an easy, practical, painless, non-invasive alternative to oral administration and required less patient cooperation [12-14]. The consequences of intranasal midazolam were later overcome by stabilizing it by storage in a 3.3 pH solution [15,16].

Early methods of intranasal midazolam sedation included drops. Still, more recently, intranasal administration with an atomizer has gained popularity, as the MAD prevents the backflow of the drug. To optimize the advantages of intranasal sedation and address potential limitations of nitrous oxide-oxygen sedation, a study was conducted to assess parents' understanding of this technique. Therefore, the purpose of this study was to ascertain the parent's and children's opinions, beliefs, and understanding of intranasal sedation and the MAD's mode of drug delivery.

## Materials and methods

Study design

The study was conducted at Saveetha Dental College and Hospital in Chennai from January 2023 to May 2023. Prior ethical approval for the study was obtained from the institute's ethical committee (IHEC/SDC/PEDO-2103/23/106). The informed consent was obtained from the parents of the children who participated in the study. The sample size was calculated from an initial pilot study and arrived at a total sample of 50. The mentioned sample size in the study was calculated according to the following formula: n = 2SP^2^[ Z_1- α__/2 _+ Z_1 -β_]^2^; Sp^2^= S_1_^2^+S_2_^2^ ^μ_d_2^ / 2 wherein S_1_ = Standard deviation of Group 1, S_2 _=Standard deviation of group 2, μ_d_^2^ = Mean difference between the samples, α = Level of significance and 1-β = Power.

Inclusion criteria

Children are categorized as ASA 1 (American Society of Anesthesiologists - physical status classification system). Children with a negative score (2) on Frankl's behavior rating scale. Patients who have not responded to basic non-pharmacological behavior guidance techniques. Children who had a single dental procedure requiring local anesthetic administration such as extraction, pulp therapy, or crown preparation were taken in for the study. Fifty parents aged 20-50 years and children aged five to nine years were involved in the study.

Exclusion criteria

Children who lack cooperative ability. Children with underlying systemic diseases or known allergies. Children with special healthcare needs. Children who were administered analgesics six hours prior to the procedure. Parents who spoke languages other than English and Tamil were excluded.

Pre-operative assessment

A comprehensive oral examination was conducted on the initial visit. The individuals underwent an airway patency exam prior to each trial to ensure there was no upper respiratory illness present and that the person could breathe comfortably through their nose. After assessment by the anesthetist, the required treatment was done on the second visit. The American Academy of Pediatric Dentistry's fasting procedure was strictly adhered to.

Clinical procedure

On the procedure day, an initial preview video of the intranasal sedation procedure was shown to the parents and children separately. A questionnaire to assess the perception of parents and children toward MAD was developed in English and Tamil languages. The questionnaire was pretested within the clinic among 15 people to ensure that the participants could understand the questions and respond in a consistent manner. The questionnaire regarding the perception of intranasal MAD was filled out by parents and the children with the help of a dentist. The child was taken to the dental operatory and positioned in a supine posture on the dental chair. A pulse oximeter probe was fitted on the index finger, which allowed for continuous monitoring of physiological measures like heart rate and hemoglobin oxygen saturation. The behavior of the child before the start of treatment was noted by the operator in an information sheet. The behavior of the child was evaluated using the Frankl behavior rating scale (Table 1) [1].

**Table 1:** Frankl behavior rating scale

Rating	Attitude	Definition
1	Definitely negative	Refusal of treatment, crying forcefully,fearful or any other overt evidence of extreme negativism
2	Negative	Reluctant to accept treatment,uncooperative,some evidence of negative attitude but not pronounced ie. sullen/withdrawn
3	Positive	Acceptance of treatment,at times cautious,willingness to comply with the dentist,at times with reservation but follows the dentist’s directions cooperatively
4	Definitely positive	Good rapport with the dentist ,interested in the dental procedures,laughing and enjoying the situation

The indicated treatment under local anesthetic administration was completed with the help of intranasal sedation by a single operator in the presence of an anesthetist. The operator was pre-trained in providing intranasal sedation to children. The intranasal drug used was midazolam (0.3 mg/kg) (Mezolam 5 g/mL, Neon Laboratories Ltd, Mumbai, India). Behavior was assessed after the completion of treatment and noted by the operator. The total duration of the procedure lasted from 15 to 45 minutes. The first sign of recovery which included movement of the limbs was seen after 60 to 80 minutes. Every child was kept under observation for two hours after treatment, adhering to the post-operative fasting protocol. All the participants became fully conscious after 80 minutes. Supplemental oxygen was not given to any of the patients, as none of them exhibited any signs of respiratory depression.

Statistical analysis

All statistical analyses were done with the SPSS Version 23 (IBM Corp., Armonk, NY). The level of significance was set to be p<0.05. The chi-square test was used to analyze the completed questionnaires. A paired t-test was used for behavior assessment of the children before and after intranasal sedation.

## Results

Out of the 63 parents who agreed to participate in the study, 50 parents returned fully completed questionnaires, providing answers to all acceptability questions. The results presented in this report are derived from this group of 50 parents. The average age of the parents included was 36 years. Within this group, 30 parents (60%) were identified as female, and 20 parents (40%) as male (Table 2).

**Table 2:** Descriptive statistics

Age		Gender	
Mean ± Standard Deviation		N (%)	
Parents	36.2 ± 2.132	Male	20 (40%)
		Female	30 (60%)
Children	9.16 ± 1.781	Boys	22 (45%)
		Girls	28 (55%)

Table 3 showcases the choices opted by the parents from the questionnaire. 

**Table 3:** Questionnaire for parents

S.No	Question	Choice	n (%)
1	Age of parents	20-30	9 (18 %)
		30-40	26 (52%)
		40-50	15 (30%)
2	Level of education	Less than high school	2 (4%)
		High school	4 (8%)
		Graduate	41 (82%)
		Post-graduate	3 (6%)
3	Socioeconomic status	Low	9 (12%)
		Medium	38 (76%)
		Upper	11 (22%)
4	Have you undergone dental treatment in the past	Yes	34 (68%)
		No	16 (32%)
5	If yes , was the experience bad	Unattempted	18 (35%)
		Yes	21 (43%)
		No	11 (22%)
6	Has the child undergone dental treatment before?	Yes	19 (38%)
		No	31 (62%)
7	If yes , was the experience bad ?	Unattempted	18 (36%)
		Yes	21 (42%)
		No	11 (22%)
8	Has the child undergone treatment under sedation in the past?	Yes	18 (36%)
		No	32 (64%)
9	After hearing the dentist explain the procedure,do you find the procedure to be safe?	Yes	26 (52%)
		No	24 (48%)
10	Will you prefer to get your child treated under intranasal sedation?	Yes	48 (96%)
		No	2 (4%)
11	If yes,will you prefer to stay with your child during the procedure	Yes	50 (100%)
		No	0
12	Is it acceptable for you,if your child sleeps during the procedure?	Yes	35 (70%)
		No	15 (30%)
13	Is it acceptable to you,if the child screams/cries during the procedure?	Yes	96 (92%)
		No	4 (8%)
14	If yes,will you accept that the treatment is accomplished ?	Unattempted	0
		Yes	50 (100%)
		No	0
15	Would you like your child to get treatment under intranasal sedation ?	Yes	47 (94%)
		No	3 (6%)

Table 4 showcases the choices opted by the children from the questionnaire. 

**Table 4:** Questionnaire for children

S.No	Question	Choice	n (%)
1	What is your age?	5-8	12 (24%)
		9-11	38 (76%)
		12-14	0
2	Have you done any dental treatment earlier?	Yes	19 (38%)
		No	31 (62%)
3	If yes,was it traumatic/painful?	Unattempted	21 (42%)
		Yes	20 (39%)
		No	9 (19%)
4	Did you like what the doctor explained to you ?	Yes	50 (100%)
		No	0
5	Would you like to get your treatment done in the same way ?	Yes	47 (94%)
		No	3 (6%)
6	Will you be scared to get this procedure done ?	Yes	45 (90%)
		No	5 (10%)

When inquired about their highest level of educational attainment, a significant portion of the parents, specifically 41 individuals (82%), reported having earned a graduation degree. A smaller percentage, three parents (6%), indicated completing post-graduation, while four parents (8%) reported the completion of high school, and two parents (4%) disclosed that they had completed less than the sixth grade. Each parent's social status was determined according to the criteria outlined by Muhammad et al., which takes into account factors such as age, gender, education, nationality, and socioeconomic status [17].

The majority of the patients included in this study were accompanied by their mothers, who were primarily in their third decade of life (52%). Only 4% of parents did not prefer intranasal sedation, and this group primarily consisted of parents aged 20-40 years, falling under the middle-class socioeconomic status. The association statistics of parental acceptance, socioeconomic status, educational status, and child's past experience are depicted in Table 5.

**Table 5:** Association between parental acceptance of MAD, socioeconomic status, educational status, and child’s past dental treatment using chi-square test. *p<0.05 statistically significant MAD - mucosal atomization device

Variable	Sub variable	Classification	n(%)	P value
Parental acceptance	Socioeconomic status	Low	9(12%)	0.000*
		Middle	38(76%)	
		Upper	11(22%)	
	Educational status	Less than high school	2(4%)	0.000*
		High school	4(8%)	
		Graduate	41(82%)	
		Post graduate	3(6%)	
	Child has undergone dental treatment in the past	Yes	19(38%)	0.038*
		No	31(62%)	

Parents of middle socioeconomic status showed a higher level of acceptance towards treatment using MAD (P = 0.000). The Chi-square test revealed that parental education also displayed a significant association with their acceptance of this sedation technique, with higher education levels correlating with greater parental acceptance (P = 0.000). Parents who had attained postgraduate qualifications were more accepting of their child's potential to sleep, cry, or scream during the dental procedure. Parents whose children had prior dental experiences (42% of the total) were more likely to view intranasal sedation as a safe option and expressed their willingness to have their child undergo treatment under similar conditions. This finding was statistically significant (P = 0.038). Notably, 36% of the surveyed children had been previously sedated, and a significant 96% of them agreed to receive treatment using intranasal sedation. The behavior assessment of children before and after treatment is depicted in Table 6.

**Table 6:** Assessment of behavior under intranasal sedation using paired t-test *p<0.05 statistically significant

Before sedation n(%)		After treatment completion with sedation n (%)		P-value
Frankl’s behavior rating 2	50 (100%)	Frankl’s behavior rating 1	2 (4%)	0.000*
		Frankl’s behavior rating 2	4 (8%)	
		Frankl’s behavior rating 3	43 (86%)	
		Frankl’s behavior rating 4	1 (2%)	

The behavior of the children was evaluated both before initiating the treatment and after concluding the treatment involving intranasal sedation with midazolam, utilizing the Frankl behavior rating scale. Participants belonging to Frankl's behavior rating 2 were included in the present study. After completion of treatment, the majority of the participants showcased positive behavior - Frankl's behavior rating 3 (n=43, 86%). On statistical analysis using paired t-test, a notable improvement in behavior was observed. This difference was found to be highly significant (P = 0.000).

## Discussion

In the current study, children between the ages of five and nine years were included. Children within this age span usually have primary teeth, which might necessitate various dental procedures like restorations, extractions, or pulp therapy. This phase often marks the beginning of dental anxiety or fear due to initial dental experiences. The dynamic relationship between children, parents, and dentists is intricate and multifaceted. Parents play a pivotal role in deciding treatment approaches and how these treatments are administered [18]. Concurrently, a child's perceptions about oral health are greatly influenced by parental words and actions [19]. Dentists, in ensuring successful treatments, utilize various methods to manage children's behavior during dental procedures. The use of intranasal sedation is contraindicated in cases of hypersensitivity reactions, otorhinolaryngeal conditions such as rhinitis or nasal polyposis, respiratory diseases, cardiac diseases, and upper respiratory tract infections. Additionally, recent administration of nasal vasoconstrictors may reduce the absorption of intranasally administered medications [19].

Identifying acceptable treatment techniques among parents and understanding the factors influencing their approval or disapproval of specific methods is crucial. Contemporary trends indicate a growing openness among many parents to move away from traditional disciplinary methods in favor of pharmacological approaches.

Research by Eaton et al. highlighted parental acceptance of pharmacological BMTs, ranking nitrous oxide inhalation sedation higher than general anesthesia [20]. However, global trends in parental acceptance of pharmacological BMTs vary. In Jordan, techniques like “tell-show-do,” positive reinforcement, and distraction received strong parental approval, contrasting with lower acceptance levels for “hand-over-mouth,” nitrous oxide, conscious sedation, and general anesthesia [21]. When comparing the acceptance rates of pharmacological and non-pharmacological behavior management techniques, it was found that non-pharmacological methods were more widely accepted [20]. No prior research has been conducted to assess the acceptability of intranasal sedation.

In this context, a study by Bhandari et al. explored parental opinions, attitudes, and knowledge regarding conscious sedation and mask acceptance while delivering nitrous oxide sedation [22]. While generally perceiving sedation as safe, Indian parents displayed limited awareness about intranasal sedation using MAD as a BMT. The present study suggests that socioeconomic status may influence the acceptance of intranasal sedation using MAD as a BMT. Individuals from middle and upper socioeconomic backgrounds tended to show greater acceptance of this approach. In our study, 96% of parents preferred intranasal sedation, with surprising readiness shown by over 50% of parents upon the dentist's recommendation. Notably, even older parents were more open to this technique. Hence, pediatric dentists should take the lead in explaining the benefits of intranasal sedation as a viable BMT. Proactively recommending intranasal sedation, when appropriate, can improve parental understanding and acceptance of this method. The study emphasizes the importance of MAD-based intranasal sedation in pediatric dentistry, especially for children uncomfortable with traditional sedation masks. MADs, being smaller and non-invasive, offer a more comfortable alternative, ensuring better patient cooperation during pediatric sedation.

The study has certain limitations that need to be acknowledged. One of the primary limitations is that the observed variations in behavior are likely to be influenced by the specific sedative drug used. Different drugs may yield different behavioral outcomes, and behavior can either improve or deteriorate based on the particular drug administered. These variations emphasize the need for careful consideration of drug selection and highlight the potential impact on behavioral responses in pediatric patients. A few other limitations were noted in the study, including the limited sample size, absence of post-treatment assessment, restricted external validity, and the lack of objective assessment tools. Comparing the depth of sedation provided by MAD with other modes of drug delivery is essential to accurately assess the efficacy of MAD. This comparison would help determine whether MAD achieves comparable levels of sedation as other methods and provide valuable insights into its effectiveness in clinical practice. These limitations may impact the generalizability and reliability of the results, and future research with larger sample sizes and comprehensive assessment measures is warranted to further explore the acceptability and efficacy of intranasal sedation in dental settings.

## Conclusions

Despite the prevalence of oral and intravenous sedation in pediatric settings, the adoption of MAD remains a viable alternative due to its painless and minimally invasive characteristics. Other methods of intranasal sedation, such as using a dropper, may encounter issues with drug loss due to backflow of the solution after administration. This concern is mitigated by MAD, as its cushion-like design covers the nostrils, preventing such loss and ensuring effective drug delivery. This research successfully explored the perspectives of both parents and children regarding intranasal sedation delivered through the MAD. By examining these perspectives, the study made a significant contribution to understanding the acceptability of using this particular method for sedation during pediatric dental procedures. The findings highlighted a positive inclination and trust from parents towards intranasal sedation facilitated by MAD, underscoring its potential as a well-received and reliable approach in pediatric dental care.

## References

[1] Sharma A, Tyagi R (2011). Behavior assessment of children in dental settings: a retrospective study. Int J Clin Pediatr Dent.

[2] Senthil Eagappan AR, Nagappan N, Senthil D, Jayanthi K, Dhanalakshmi V, Sowmiyasree RA, Narayanan B (2021). Paediatric dentists' knowledge of behavioural management principles in Tamil Nadu, India. Eur J Paediatr Dent.

[3] Baakdah RA, Turkistani JM, Al-Qarni AM, Al-Abdali AN, Alharbi HA, Bafaqih JA, Alshehri ZS (2021). Pediatric dental treatments with pharmacological and non-pharmacological interventions: a cross-sectional study. BMC Oral Health.

[4] Gao F, Wu Y (2023). Procedural sedation in pediatric dentistry: a narrative review. Front Med (Lausanne).

[5] Harbuz DK, O'Halloran M (2016). Techniques to administer oral, inhalational, and IV sedation in dentistry. Australas Med J.

[6] Cloyd J, Haut S, Carrazana E, Rabinowicz AL (2021). Overcoming the challenges of developing an intranasal diazepam rescue therapy for the treatment of seizure clusters. Epilepsia.

[7] Janiani P, Gurunathan D, Manohar R (2023). Assessment of pain during pediatric dental treatment using different sedative agents: a crossover trial. Cureus.

[8] Becker DE, Rosenberg M (2008). Nitrous oxide and the inhalation anesthetics. Anesth Prog.

[9] Peerbhay F, Elsheikhomer AM (2016). Intranasal midazolam sedation in a pediatric emergency dental clinic. Anesth Prog.

[10] Isik B, Baygin O, Bodur H (2008). Effect of drinks that are added as flavoring in oral midazolam premedication on sedation success. Paediatr Anaesth.

[11] Kotian N, Subramanian EM, Jeevanandan G (2022). Comparing the sedative effect of oral and intranasal midazolam and their effect on behavior in pediatric dental patients. Int J Clin Pediatr Dent.

[12] Grassin-Delyle S, Buenestado A, Naline E (2012). Intranasal drug delivery: an efficient and non-invasive route for systemic administration: focus on opioids. Pharmacol Ther.

[13] Goyal AK, Singh R, Chauhan G, Rath G (2018). Non-invasive systemic drug delivery through mucosal routes. Artif Cells Nanomed Biotechnol.

[14] Ann Preethy N, Somasundaram S (2022). Safety and physiologic effects of intranasal midazolam and nitrous oxide inhalation based sedation in children visiting Saveetha Dental College and Hospitals, India. Bioinformation.

[15] Raj S, Agarwal M, Aradhya K, Konde S, Nagakishore V (2013). Evaluation of dental fear in children during dental visit using children’s fear Survey Schedule-dental subscale. Int J Clin Pediatr Dent.

[16] Preethy NA, Somasundaram S (2021). Edative and behavioral effects of intranasal midazolam in comparison with other administrative routes in children undergoing dental treatment - a systematic review. Contemp Clin Dent.

[17] Muhammad S, Shyama M, Al-Mutawa SA (2011). Parental attitude toward behavioral management techniques in dental practice with schoolchildren in Kuwait. Med Princ Pract.

[18] Al-Batayneh OB, Al-Khateeb HO, Ibrahim WM, Khader YS (2019). Parental knowledge and acceptance of different treatment options for primary teeth provided by dental practitioners. Front Public Health.

[19] Chawłowska E, Karasiewicz M, Lipiak A (2022). Exploring the relationships between children’s oral health and parents' oral health knowledge, literacy, behaviours and adherence to recommendations: a cross-sectional survey. Int J Environ Res Public Health.

[20] Eaton JJ, McTigue DJ, Fields HW Jr, Beck M (2005). Attitudes of contemporary parents toward behavior management techniques used in pediatric dentistry. Pediatr Dent.

[21] Alammouri M (2006). The attitude of parents toward behavior management techniques in pediatric dentistry. J Clin Pediatr Dent.

[22] Bhandari R, Thakur S, Singhal P, Chauhan D, Jayam C, Jain T (2018). Parental awareness, knowledge, and attitude toward conscious sedation in North Indian children population: a questionnaire-based study. Indian J Dent Res.

